# Isolation of atypical enteropathogenic and shiga toxin encoding *Escherichia coli* strains from poultry in Tehran, Iran 

**Published:** 2016

**Authors:** Fatemeh Doregiraee, Masoud Alebouyeh, Bahar Nayeri Fasaei, Saeed Charkhkar, Elahe Tajedin, Mohammad Reza Zali

**Affiliations:** 1 *Department of Microbiology and Immunology, Faculty of Veterinary Medicine, University of Tehran, Tehran, Iran*; 2 *Foodborne and Waterborne Diseases Research Center, Research Institute for Gastroenterology and Liver Diseases, Shahid Beheshti University of Medical Sciences, Tehran, Iran*; 3 *Gastroenterology and Liver Diseases Research Center, Research Institute for Gastroenterology and Liver Diseases, Shahid Beheshti University of Medical Sciences, Tehran, Iran*; 4 *Faculty of Veterinary Medicine, Islamic Azad University, Science and Research Branch, Tehran, Iran*

**Keywords:** *E. coli*, O157:nonH7, STEC, Atypical EPEC, Poultry

## Abstract

**Aim:**

The purpose of this study was to investigate the prevalence of enteropathogenic *Escherichia coli* (EPEC) and shiga toxin producing *E. coli* (STEC) strains in healthy broilers in Iran.

**Background:**

STEC and EPEC strains as diarrheagenic *E. coli* are among the most prevalent causative agents in acute diarrhea. Domestic animals, mainly cattle and sheep, have been implicated as the principal reservoirs of these pathotypes; however their prevalence among the broilers is varied among different countries.

**Patients and methods:**

A total of 500 cloacal swab samples from broilers of five different poultry houses (A-E) were collected to investigate the presence of *stx1*, *stx2*, *hly*, *eae*, and *bfp* virulence genes among the *E. coli* isolates by polymerase chain reaction. The shiga toxin encoding strains were evaluated serologically to detect their interaction with a commercial antiserum against O157 antigen.

**Results:**

Out of the 500 collected samples, 444 *E. coli* strains were isolated. Three strains (0.67%) presented at least one of the studied virulence genes (*stx2*, *hly* and *eae*), two strains were identified as STEC (*stx2*^+^, O157:nonH7) and one as an atypical EPEC strains (*eae*^+^ *bfp*^-^).

**Conclusion:**

The study established the presence of STEC and atypical EPEC in healthy broilers in Iran. Poultry might serve as vectors for transmission of pathogenic *E. coli* to human populations.

## Introduction

 Shiga toxin-producing *Escherichia coli* (STEC) and enteropathogenic *E. coli* (EPEC) are considered as two diarrhoeagenic *E. coli* pathotypes (1). STEC, a serologically diverse group of foodborne zoonotic pathogens, is an important causative agent of haemorrhagic colitis (HC) and diarrhoea-associate haemolytic uremic syndrome (HUS) with or without neurological complications (2). Animals, especially ruminants (cattle, sheep and goat) have been implicated as the reservoirs of STEC (3). Two phage- encoded cytotoxins (Stx1 and Stx2), which are produced by *stx1* and *stx2* genes, mediate mainly the pathogenicity of STEC. The virulence-associated factor intimin, encoded by *eae*, is responsible for intimate attachment of STEC to the enterocytes, causing attaching and effacing (A⁄E) lesions in the intestinal mucosa (4). Serotype O157:H7 has been the predominant type worldwide (5). EPEC strains, as intimin-containing diarrhoeagenic *E. coli*, possess the ability to form attaching-and-effacing (A⁄E) lesions on intestinal cells (6). Based on the presence or absence of bundle forming pili gene (*bfpA*), EPEC strains are classified as typical and atypical (7). There are several reports of the prevalence and characterization of STEC in poultry (1, 8) and wild birds, including pigeon (9). However, in Iran, such information from avian species is scant. Therefore, the aim of the present study was to study the prevalence of STEC and EPEC in avian species, to analyze the *stx* variants (*stx1* or *stx2*) and the typical and atypical nature of the EPEC. 

## Patients and Methods

**Bacterial isolates**

A total of 500 cloacal swab samples from avian of five different poultry houses were collected over the six-month period in 2013 in Tehran, Iran. Samples were transported to the laboratory in the Cary- Blair medium (Merck, Germany). For *E. coli* isolation, the swabs were immediately inoculated into MacConkey agar medium (Merck, Germany). The plates were incubated for 18–24 h at 37 °C. Single colonies with typical color and appearance of *E. coli* were isolated from each sample for further analysis. Characterization of the suspected *E. coli* isolates was performed according to conventional laboratory biochemical tests (10).

**PCR for detection of** ***stx1, stx2, eae and bfp*** **genes**

Primers used in the study are listed in Table 1. The template DNA was prepared as described by the method of Blanco et al. (11). Avian *E. coli* isolates were subjected to PCR for detection of *stx1, stx2, hly, eae* and *bfp *genes.

**Phenotypic identification of** ***E. coli*** **O157 strains**

Ability of sorbitol fermentation of Shiga toxin encoding strains was evaluated on Sorbitol MacConkey agar medium. A Commercial serologic test was used for the detection of O157 serotype (Baharafshan, Iran) according to the manufacturers’ instruction manual (17). 

## Results

**Figure 1:**
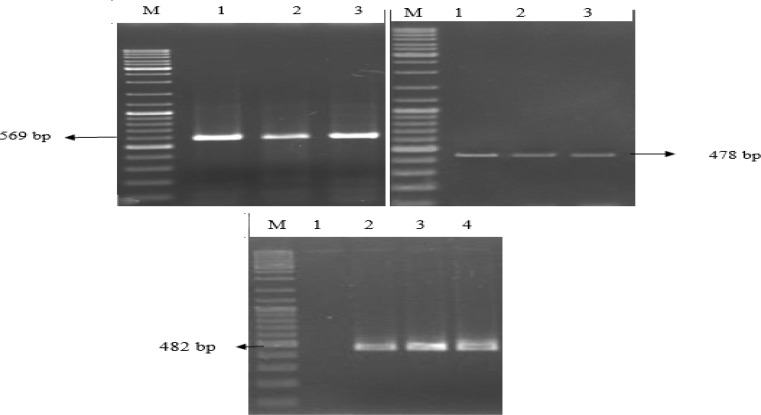
Detection of *hly* (top left), *stx2 *(top right)*,* and *eae* (bottom) genes in *E. coli* isolates from poultry. Lane M: DNA ladder mix, Lane 2: Positive control for each genes

Out of the 500 collected samples, about 88.8% of the samples showed positive culture results for *E. coli*. Most of the isolates were related to lactose positive *E. coli* strains (98.6%), therefore the poultry house E included the most *E. coli* isolate lactose negative (3/6). Three of 444 *E. coli* isolates showed the presence of one of the studied virulence genes, among them two strains belonged to STEC and one to EPEC. Both of two STEC isolates were harbored *stx2* and *hly*, but lacked *eae* virulence gene. None of the *eae*^+ ^EPEC isolates harbored *bfp* and were designated as atypical (*eae*^+^/*bfp*^-^) (Figure 1). Serotyping of the Shiga toxin encoding strains showed that they belonged to O157 serotype and were identified as O157:nonH7 strains. While the EPEC strain showed a positive reaction with the sorbitol fermentation test, both the STEC strains failed to ferment sorbitol. 

**Table1:** Nucleotide sequences of primers that used in PCR for detection of *E. coli* pathotypes

Target gene	Primer	Sequence(5'-3')	Size (bp)	Reference
*stx1*	Stx1F	5׳ GAAGAGTCCGTGGGATTACG	130	(12)
	Stx1R	5׳ AGCGATGCAGCTATTAATA		
*stx2*	Stx2F	5׳ GGATGCATCTCTGGTCATTG	478	(13)
	Stx2R	5׳ CTTCGGTATCCTATTCCCGG		
*hly*	HlyF	5׳ AGCTGCAAGTGCGGGTCTG	569	(14)
	HlyR	5׳ TACGGGTTATGCCTGCAAGTTCAC		
*eae*	EaeF	5 ׳TCAATGCAGTTCCGTTATCAGTT	482	(15)
	EaeR	5׳ GTAAAGTCCGTTACCCCAACCTG		
*bfp*	BfpF	5׳ CACCGTTACCGCAGGTGTGA	450	(16)
			

## Discussion

EPEC and STEC are foodborne pathogens that produce potentially fatal infant diarrhea and bloody diarrhea / hemolytic uremic syndrome, respectively (18, 19). In the present study, two and one strains of *E. coli* from cloacal swabs of broilers identified as STEC and atypical-EPEC, respectively. Transmission of these pathogenic *E. coli* strains from poultry to human through consumption of inadequate cooked products is problematic. Compared with studies that performed on cattle and sheep, limited numbers of surveys analyzed the prevalence of STEC and EPEC from avian *E. coli* pathotypes (20, 21). Contrary of data exist to correlate carriage of pathogenic *E. coli* in poultry and infection of human. In a study that was performed in Iran in 2014, EPEC and STEC pathotypes were detected from 12 carcasses of broiler chickens (22). Farooq et al. showed the presence of these pathotypes in avian species in India. Among 212 faecal samples, nine (4.24%) isolates were STEC, and 33 (15.56%) were EPEC (23). 

However, the absence of STEC in chicken was established in different studies (24-26). It has already been established that typical EPEC are rarely isolated from animals, while atypical EPEC strains are isolated from both animals and humans. Detection of atypical EPEC in the present study is in agreement with the observations of Krause et al. who reported the presence of atypical EPEC in poultry in Germany (28). We reported that two STEC strains possessed *stx2*, which is consistent with the finding of Ghanbarpour et al. (2011) who revealed that 4.5% from healthy broilers in Iran harbored *stx2 *(29). Detection of *stx2* in our study is in accordance with the observations of other investigations in pigeon who reported the isolation of *Stx2* producing *E. coli* with the exception that belonged to O45, O18 and O75 serogroups (30, 31). However, in our study these strains belonged to serogroup O157. Doyle and Schoeni in 1987 reported the presence of *E. coli* O157 in poultry meat for the first time and stated that the organism is not a rare contamination of fresh meat and poultry (32). Similarly, verotoxin producing *E. coli* strains of serogroup O157 were detected among poultry in the Netherlands (33).

 To the best of our knowledge, this is the first report of the presence of *E. coli* O157 serogroupe in healthy broilers in Iran. Seeking the environmental sources of these infections was the main limitation of this study that should be considered for a better control of their transmission.

In conclusion, our findings provide an initial data about the carriage of STEC and atypical-EPEC in healthy poultry in Iran. Therefore, poultry might serve as vectors for transmission of pathogenic *E. coli* strains to the human populations. The study of EPEC and *E. coli* O157 contamination at different stages of the slaughtering process in a chicken processing plant is a practical suggestion for more precise tracking of foodborne pathogens in the human food chain.
